# 高效液相色谱-四极杆飞行时间质谱法测定液态乳中糠氨酸

**DOI:** 10.3724/SP.J.1123.2023.05009

**Published:** 2023-11-08

**Authors:** Guihong YAO, Yun LING, Yujia ZHANG, Shige XING, Meiyi YAO, Wei GUO, Feng ZHANG

**Affiliations:** 中国检验检疫科学研究院食品安全研究所, 北京 100176; Institute of Food Safety, Chinese Academy of Inspection and Quarantine, Beijing 100176, China

**Keywords:** 高效液相色谱-四极杆飞行时间质谱法, 高分辨质谱, 糠氨酸, 液态乳, high performance liquid chromatography-quadrupole time-of-flight mass spectrometry (HPLC-Q-TOF/MS), high resolution mass spectrometry (HRMS), furosine, liquid milk

## Abstract

国内外常使用糠氨酸含量作为牛奶受热程度的指标用于评价牛奶品质,但在实际检测时,由于牛奶基质复杂可能造成液态乳液中糠氨酸定量不准,因此,本研究基于高效液相色谱-高分辨四极杆飞行时间质谱法建立了高效准确的液态乳中糠氨酸的检测方法。取2.00 mL牛奶样品,加入5 mL 12 mol/L盐酸溶液和1 mL水后在110 ℃条件下水解12 h,水解完成后涡旋混匀过滤,滤液经6.00 g/L的乙酸铵溶液稀释6倍后进样分析,以0.20%甲酸水溶液和乙腈溶液为流动相进行梯度洗脱,AQ-C_18_色谱柱(150 mm×3.5 mm, 5 μm)进行分离,在高分辨四极杆飞行时间质谱仪的电喷雾电离正离子模式下采集数据。为确保牛乳中糠氨酸定量的准确性,考察了糠氨酸溶液中盐酸浓度(0.30、1.25、3.00 mol/L)对质谱响应的影响,结果显示,盐酸浓度过高会抑制质谱响应信号。糠氨酸在0.05~2.00 mg/L范围内线性关系良好,相关系数(*r*)为0.994,方法检出限为0.50 mg/100 g,满足实际样品检测需求。在1.52、3.03、15.17 mg/100 g 3个添加水平下对糠氨酸进行加标回收试验,平均回收率为79.9%~119.7%,相对标准偏差(RSD, *n*=6)为1.4%~2.6%。应用该方法对某市售巴氏杀菌乳101批次303个样品进行检测,样品中糠氨酸含量在5.1~11.9 mg/100 g之间。该方法高效快速,回收率高,灵敏度好,分析准确,可进行大批量的样品测定,为持续推动奶业全产业链高质量发展提供技术支持。

## Abstract (en)

Furosine is often used both domestically and internationally as an indicator of the degree of heating to evaluate milk quality. However, in actual detection, the complexity of the milk matrix may lead to the inaccurate quantification of furosine in liquid milk. Therefore, in this study, an efficient and accurate method based on high performance liquid chromatography-quadrupole time-of-flight mass spectrometry (HPLC-Q-TOF/MS) was established to determine furosine in liquid milk. A 2.00 mL milk sample was hydrolyzed with 5 mL 12.00 mol/L hydrochloric acid solution and 1 mL water at 110 ℃ for 12 h. After hydrolysis, vortex-mixing and filtration were performed. The filtrate was diluted six times with 6.00 g/L ammonium acetate solution and then analyzed. Gradient elution was performed with 0.20% formic acid aqueous solution and acetonitrile solution as mobile phases, followed by chromatographic separation on an AQ-C_18_ column (150 mm×3.5 mm, 5 μm). The data were collected by Q-TOF/MS with an electrospray ionization source operated in positive-ion mode. The accuracy of the quantification of furosine in milk was assessed by investigating the effects of the hydrochloric acid concentration (0.30, 1.25, and 3.00 mol/L) in the furosine solution on the MS response. The results showed that high hydrochloric acid concentrations inhibited the response signals. A good linear relationship was obtained in the mass concentration range of 0.05-2.00 mg/L, with a correlation coefficient (*r*) of 0.994. The limit of detection of the method was 0.50 mg/100 g, which meets the requirements of actual sample detection. The average recoveries of furosine ranged from 79.9% to 119.7% at three spiked levels of 1.52, 3.03, and 15.17 mg/100 g, with relative standard deviations of 1.4%-2.6%. The method was applied to detect 303 samples from 101 batches of pasteurized milk sold in the market, and the contents of furosine in these samples ranged from 5.1 to 11.9 mg/100 g. The proposed method is characterized with high efficiency, recovery, sensitivity, and accuracy. Thus, it can be used for the determination of large quantities of samples and provides technical support for the continuous promotion of the high-quality development of the whole dairy industry chain.

牛奶在进入市场前一般需要经过热处理来减少微生物数量,延长牛奶的保质期^[1⇓⇓-4]^。然而牛奶在热处理或贮存过程中极易发生美拉德反应^[1,5,6]^,这对牛奶的营养价值、理化性质和风味均会产生较大的影响,甚至诱发生成糠醛、晚期糖基化终末产物、丙烯酰胺等对人体健康有害的产物^[7⇓⇓-10]^,且这些有害产物在存储期间可能会进一步增加,因此需要选择合适的热敏指标来评判牛奶的热处理强度。糠氨酸是乳品美拉德反应的初级阶段产物Amadori化合物酸水解时形成的产物^[11,12]^,化学性质较Amadori化合物更稳定^[13]^。由此可以用糠氨酸间接量化Amadori产物,来评估牛乳的美拉德反应程度。国际上常用糠氨酸含量作为判断液态奶产品品质的一个敏感指标^[14⇓⇓⇓-18]^。其中,欧盟各国早在1992年就将糠氨酸作为评判乳制品品质的一个重要指标,并于1996年提出用糠氨酸作为判定复原乳的主要指标。为提高我国乳业的核心竞争力,我国发起并实施了优质乳工程,并通过对国产奶和进口奶科学系统的对比研究,确定了牛乳中糠氨酸可以作为品质高低的评判指标^[19]^。

目前常见的糠氨酸检测方法主要有高效液相色谱法(HPLC)^[12,19,20]^、超高效液相色谱法^[21,22]^、液相色谱-串联质谱法^[23⇓-25]^、高效液相色谱-四极杆飞行时间质谱法(high performance liquid chromatography-quadrupole time-of-flight mass spectrometry, HPLC-Q-TOF/MS)^[26]^、毛细管电泳-串联质谱法^[27]^和表面荧光法^[28]^等。其中,HPLC是应用最早、普及最广的检测方法,国际奶业联合会(International Dairy Federation, IDF)和国际标准化组织(International Organization for Standardization, ISO)于2014年颁布的乳及乳制品中糠氨酸含量的测定方法(ISO 18329-2004)^[29]^及我国农业行业标准NY/T 939-2016《巴氏杀菌乳和UHT灭菌乳中复原乳的鉴定》中均采用了HPLC。但在实际检测时,由于牛奶水解样品中含有糖、有机酸等杂质,可能会严重干扰糠氨酸的准确定量。相较常用的HPLC,液相色谱-串联质谱法和HPLC-Q-TOF/MS抗干扰能力强,在排除干扰组分鉴别目标物方面具有优势,非常适合检测复杂牛奶基质中的糠氨酸。糠氨酸是酸水解产物,糠氨酸中的酸溶液会对质谱响应有影响,进而影响糠氨酸的准确定量,尤其当糠氨酸含量较低时,易导致检测结果被误判,进而影响巴氏杀菌乳的等效性评价。现有报道的质谱法均未考察酸对质谱响应的影响。故本研究基于HPLC-Q-TOF/MS建立了液态乳中糠氨酸含量的测定方法,并考察了糠氨酸溶液中酸浓度对质谱信号的影响,为液态乳中糠氨酸的准确定量提供高效可靠的技术手段。

## 1 实验部分

### 1.1 仪器、试剂与材料

ACQUITY UPLC型超高效液相色谱系统+Xevo G2 Q-TOF型高分辨四极杆飞行时间质谱仪,配置标准电喷雾离子源(electrospray ionization, ESI)(美国Waters公司); Vortex-KB3涡旋混合器(美国Scientific Industries公司); DKN612C烘箱(重庆雅马拓科技有限公司); Advantage A10 Milli-Q去离子水发生器(美国Millipore公司); AQ-C_18_色谱柱(150 mm×3.5 mm, 5 μm,日本岛津公司)。

牛奶样品(市售,101个批次303个样品);糠氨酸标准品(纯度99.99%,美国Sigma Aldrich公司);乙酸铵、乙腈、甲酸(色谱纯,美国Thermo Fisher Scientific公司);盐酸(分析纯,国药集团化学试剂有限公司)。实验用水采用Millipore纯水仪制备。

### 1.2 实验条件

#### 1.2.1 标准溶液配制

标准储备液:称取适量糠氨酸标准品,用浓度为3.00 mol/L的盐酸配制成质量浓度为10.00 mg/L的标准储备液。

标准工作液:移取一定量的标准储备液,用1.00 mol/L的盐酸稀释至所需浓度,标准工作液中盐酸最终浓度为1.25 mol/L,临用现配。

#### 1.2.2 样品水解

王峰恩等^[11]^报道,牛乳酸解时盐酸浓度≥6 mol/L时Amadori化合物完全酸水解为糠氨酸。本研究移取2.00 mL牛奶样品置于耐热玻璃试管中,加入5 mL盐酸溶液(12.00 mol/L)和1 mL水,涡旋混匀。密闭试管并将其置于烘箱中,在110 ℃下水解12 h。加热水解结束后将耐热玻璃试管从烘箱中取出,冷却至室温,涡旋混匀后过滤。移取1.00 mL滤液,加入5.00 mL 6.00 g/L的乙酸铵溶液(此时水解液中盐酸浓度约为1.25 mol/L),混匀后过0.22 μm水相滤膜,滤液供上机待测。

#### 1.2.3 样品水解液中蛋白质测定

移取2.00 mL牛奶水解液,按GB 5009.5-2016《食品安全国家标准 食品中蛋白质的测定》规定的凯氏定氮法测定样品水解液中蛋白质的含量。

#### 1.2.4 色谱条件

色谱柱:AQ-C_18_ (150 mm×3.5 mm, 5 μm);柱温:室温(25 ℃);流动相A: 0.20%甲酸水溶液,流动相B:乙腈;进样量:5.00 μL;流速:0.30 mL/min;梯度洗脱程序:0~0.2 min, 5%B; 0.2~10 min, 5%B~100%B; 10~13 min, 100%B~5%B; 13~15 min, 5%B。

#### 1.2.5 质谱条件

在电喷雾离子源正离子模式(ESI^+^)下以全扫描方式扫描,扫描范围为*m/z* 50~400,毛细管电压4.0 kV,锥孔电压40 V,离子源温度100 ℃,去溶剂气温度350 ℃,去溶剂气流量600 L/h,为了保障质量数精度,用甲酸钠对仪器质量轴进行质量校正。

#### 1.2.6 糠氨酸含量计算

糠氨酸含量以质量分数*F*计,数值以毫克每百克蛋白质(mg/100 g)表示,按公式(1)计算:


(1) $$F=\frac{C\times D\times 100}{m}$$

式中:*C*为样品水解液中糠氨酸含量(mg/L); *D*为测定时稀释倍数(*D*=6); *m*为样品水解液中蛋白质的质量浓度(g/L)。

#### 1.2.7 基质效应考察

本研究在牛奶基质中添加糠氨酸标准溶液,配制成质量浓度为0.05、0.10、0.20、0.50、1.00和2.00 mg/L的基质标准溶液。同时配制相同质量浓度的溶剂标准溶液,以HPLC-Q-TOF/MS测定,按公式(2)计算ME:


(2) $$ME=\frac{B}{A}\times 100\%$$

其中,*B*为基质标准曲线斜率;*A*为溶剂标准曲线斜率。

当ME值为80.0%~120.0%时,表明基质对目标物干扰较小,基质效应在可接受范围内,在实际检测中可以采用溶剂标准曲线定量;反之则应采用基质匹配标准曲线降低基质干扰影响。

### 1.3 数据处理

HPLC-Q-TOF/MS所得质谱数据采用随机配置的美国Waters公司的MassLynx 4.2软件采集,在MassLynx 4.2软件上进行定性定量处理分析。

## 2 结果与分析

### 2.1 定性定量分析依据

HPLC-Q-TOF/MS测定牛奶样品中的糠氨酸时,以一级母离子的精确质量数、同位素丰度比和色谱保留时间等信息作为定性依据,母离子响应强度为定量分析依据。测定0.2 mg/L的糠氨酸标准溶液,提取离子流色谱图及质谱图见图1。糠氨酸标准品的色谱保留时间为1.23 min,精确相对分子质量为255.1333。在实际样品检测时,相应质荷比的提取离子色谱图中出现了与糠氨酸标准品保留时间一致(变化范围在±0.5%之内)的色谱峰,若精确质量数偏差范围在±5×10^-6^之内且同位素丰度比相差不大于10%,即可判断该样品中存在糠氨酸。

**图1:**
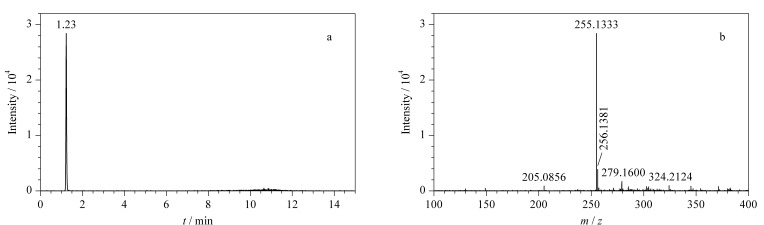
糠氨酸标准品(0.2 mg/L)的(a)提取离子流色谱图及(b)质谱图

### 2.2 盐酸浓度对糠氨酸检测结果的影响

本研究采用ESI采集质谱信号,试样中酸的浓度可能促进或抑制样品离子电离,从而影响质谱信号。因此,本研究对比了以3种不同浓度盐酸溶液(0.30、1.25、3.00 mol/L)配制的糠氨酸标准溶液的质谱响应,采集到的糠氨酸峰面积分别为2643、1359和902。随着盐酸浓度的升高,糠氨酸质谱响应降低,这可能是由于随着酸浓度增大,样品中的氢离子增多,过多的氢离子与样品离子竞争电荷,从而抑制了质谱信号。另外,糠氨酸所处溶液环境对糠氨酸的稳定性影响较大,陈美霞等^[30]^对比了糠氨酸溶解在3种不同浓度盐酸(0.1、1.0、3.0 mol/L)中的稳定性,发现用3.0 mol/L盐酸配制的糠氨酸标准溶液稳定性最佳,这是由于糠氨酸为酸性分子,较高的酸性环境有助于提高其存储稳定性。本研究实际上机样品经酸解稀释后溶液中盐酸浓度约为1.25 mol/L。为了提高目标物的定量准确性,本研究糠氨酸标准储备溶液配制在3.0 mol/L盐酸中,将标准工作液配制在1.25 mol/L盐酸溶液中,与上机试样中盐酸浓度一致。

### 2.3 流动相的选择

户江涛^[23]^对比发现,糠氨酸在酸性体系流动相中峰形更加对称,分离效果更好。多数研究在流动相中添加三氟乙酸(见表1), 三氟乙酸酸性较强,长期使用会损害色谱柱,缩短其使用寿命。本研究综合考虑目标物信号、除杂等因素, 采用酸性较弱的甲酸和洗脱能力更强的乙腈作为流动相,得到了很好的分离效果。

**表 1:** 糠氨酸检测方法比较

Mobile phases	Retention time/min	LOD/(mg/100 g)	LOQ/(mg/100 g)	Ref.
Methanol, trifluoroacetic acid	3.80	1.50	4.90	[22]
Methanol, trifluoroacetic acid	-	3.00	-	[31]
Methanol, acetic acid	3.00	2.80	-	[32]
Methanol, trifluoroacetic acid	-	1.00	3.00	NY/T 939-2016
Acetonitrile, formic acid	1.23	0.50	1.50	this method

-: not mentioned in the literature.

### 2.4 基质效应考察

牛奶样品基质复杂,样品中的其他组分在质谱分析过程中可对糠氨酸响应造成不可预期的抑制或者增强。本研究通过对比基质匹配标准曲线和溶剂标准曲线斜率来评价方法的基质效应。结果显示,溶剂标准曲线方程为*Y*=15941*X*-1650.9,基质匹配标准曲线方程为*Y*=16850*X*-2225,由此得出ME为105.70%,基质效应在可接受范围内,因此在测定过程中采用溶剂标准曲线。

### 2.5 方法的线性范围、检出限和定量限

在选定的工作参数下测定糠氨酸标准系列溶液,结果显示,在0.05~2.00 mg/L范围内,糠氨酸质量浓度与峰面积值呈良好的线性关系,线性回归方程为*Y*=15941*X*-1650.9,相关系数(*r*)为0.994。分别以信噪比为3(*S/N*=3)和10(*S/N*=10)来确定检出限(LOD)和定量限(LOQ)。LOD和LOQ分别为0.50 mg/100 g蛋白和1.50 mg/100 g蛋白。该方法的检出限与现有报道的比较见表1,相对现有报道,检出限明显降低。由于生鲜乳中的糠氨酸含量一般高于2 mg/100 g,故本方法能满足日常液态乳中糠氨酸的检测要求。

### 2.6 回收率和精密度

选取某品牌巴氏杀菌乳样品作为加标回收样品,添加低、中、高3个水平的糠氨酸标准溶液(1.52、3.03、15.17 mg/100 g)后按照1.2节方法进行样品前处理及检测,每个加标水平重复测定6次,计算平均回收率和相对标准偏差(RSD)。结果如表2所示,糠氨酸的平均回收率为79.9%~119.7%,RSD为1.4%~2.6%。表明该方法回收率高,稳定性好,能满足巴氏杀菌乳中糠氨酸的检测要求。

**表 2:** 某品牌巴氏杀菌乳中糠氨酸的加标回收率和RSD(*n*=6)

Background/ (mg/100 g)	Added/ (mg/100 g)	Found/ (mg/100 g)	Recovery/ %	RSD/ %
9.71	1.52	11.53	119.7	2.1
	3.03	12.13	79.9	2.6
	15.17	21.84	80.0	1.4

### 2.7 实际样品检测

本研究同时采用2016年发布的农业行业标准NY/T 939-2016(HPLC)和本研究所建立的HPLC-Q-TOF/MS检测某市售品牌巴氏杀菌乳中的糠氨酸,检测结果见图2。由图2a可知,采用HPLC方法,样品的糠氨酸组分与含有的杂质不能实现良好分离,进而影响定量的准确性。HPLC-Q-TOF/MS具有可以获得一级精确质量数、同位素比率信息、较完整的化合物裂解碎片信息等优势,通过提取精确的糠氨酸质荷比(质量偏差不高于5×10^-6^)可以有效去除其他组分的干扰,大大提高定性定量的准确度,且不受进样次数增加的影响。另外,本研究采用洗脱能力更强的乙腈溶液作为流动相进行检测分析,可以更好地去除分离检测系统中残留的杂质。利用所建立的HPLC-Q-TOF/MS方法检测了101个批次303个某市售品牌巴氏杀菌乳样品,市售巴氏杀菌乳的全扫描色谱图及提取精确质量数离子图如图2b~c所示。样品糠氨酸含量为5.1~11.9 mg/100 g,检测结果符合其他文献中巴氏杀菌乳中糠氨酸检测值^[16]^,间接证明本方法的可靠性。

**图2:**
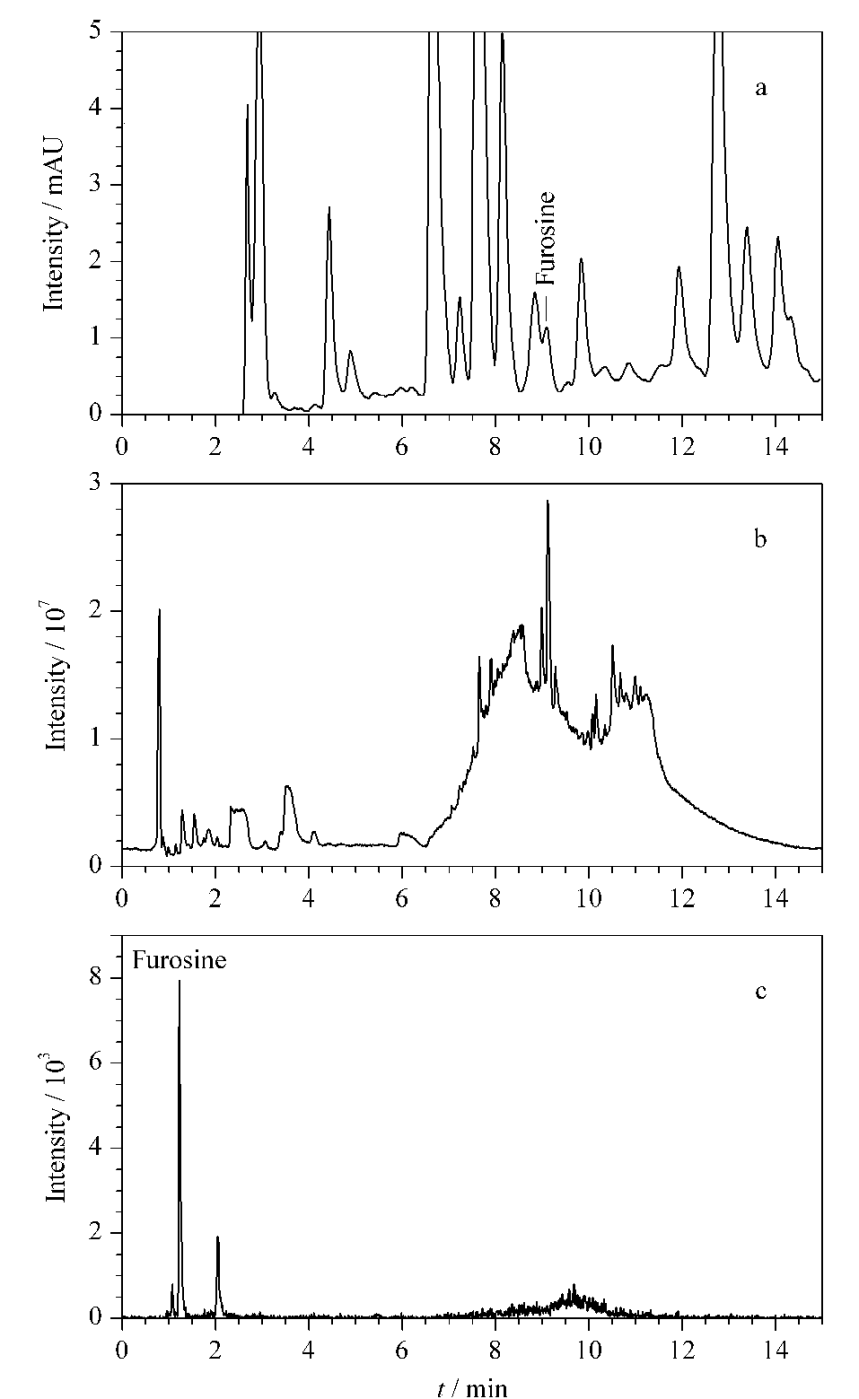
市售巴氏杀菌乳的(a)HPLC图、(b) HPLC-Q-TOF/MS全扫描图及(c)糠氨酸提取离子流色谱图

## 3 结论

糠氨酸是评价牛奶成品品质的重要指标之一。本研究针对复杂基质造成的定性定量不准确的问题,结合高分辨四极杆飞行时间质谱高效快速、高质量精度、高灵敏度的特点,建立了液态乳中糠氨酸的分析检测方法。相比于行业标准HPLC法,检测时长缩短至15 min,且通过提取精确的糠氨酸质荷比(质量偏差不高于5×10^-6^ ),并对比保留时间及同位素丰度比,可以有效去除杂质干扰,对于目标物的定性定量分析也更为精确,且不受进样次数的影响。相较现有报道的液相色谱-串联质谱法和HPLC-Q-TOF/MS法,本研究进一步考察了盐酸浓度对糠氨酸质谱响应的影响,确保糠氨酸定量更准确,实现了乳品中糠氨酸的精准检测,可用于市场牛乳中糠氨酸的监控。但本研究前处理方法仍较为复杂,今后研究中将对简化糠氨酸前处理步骤进行深入研究。
